# A retrospective study for clinical characteristics of 293 patients with dermatomyositis

**DOI:** 10.1097/MD.0000000000040605

**Published:** 2024-11-15

**Authors:** Xiaowen Xie, Xinyue Dai, Huaxiang Liu, Yi Xing

**Affiliations:** a Department of Rheumatology, Qilu Hospital, Shandong University, Jinan, China; b Department of Neurosurgery, Shandong Provincial Hospital Affiliated to Shandong First Medical University, Jinan, China.

**Keywords:** clinical features, dermatomyositis, myositis-specific antibodies, prognosis

## Abstract

This retrospective study aimed to investigate differences in clinical characteristics between different antibody phenotypes in patients with dermatomyositis (DM). Two hundred and ninety-three patients with DM were included in this study from September 2018 to September 2023. We collected basic clinical data from the patients, using statistical methods to analyze the clinical characteristics, and used survival analysis and COX regression to assess the prognosis of the patients. In the 293 patients, the antibody distribution was as follows: antibody negative (50, 20.3%), anti-melanoma differentiation-associated gene 5 (MDA5) antibody (104, 42.3%), anti-transcription intermediary factor γ (TIF-γ) antibody (41, 16.7%), anti-complex nucleosome remodeling histone deacetylase (Mi2) antibody (28, 11.4%), anti-nuclear matrix protein 2 (NXP2) antibody (19, 7.7%), anti-small ubiquitin-like modifier activating enzyme (SAE) antibody (4, 1.6%). Interstitial pneumonia (*P* < .001), lung infection (*P* < .001), respiratory symptoms (*P* < .001), arthralgia (*P* < .001), and fever (*P* < .001) were more likely to be seen in patients with anti-MDA5 antibody. Malignancy (*P* < .001) and V-sign (*P* = .017) were more likely to occur in anti-TIF1-γ antibody positive patients. Anti-NXP2 antibody-positive patients showed more symptoms of muscle involvement, such as myasthenia (*P* = .002), myalgia (*P* = .003) and dysphagia (*P* = .001). In the analysis of prognosis, age at onset (hazard ratio = 1.096, 95% CI: 1.064–1.129, *P* < .001), fever (hazard ratio = 2.449, 95% CI: 1.183–5.066, *P* = .016), γ-glutamyl transferase level (hazard ratio = 1.005, 95% CI: 1.002–1.008, *P* < .001), eosinophil level (hazard ratio = 0.000, 95% CI: 0.000–0.324, *P* = .024), and complement 3 (C3) level (hazard ratio = 0.115, 95% CI: 0.023–0.575, *P* = .008) had a statistically significant effect on survival time. The clinical features of DM are associated with myositis-specific antibodies. At the same time, advanced age, fever, elevated γ-glutamyl transferase levels, and reduced C3 and eosinophil levels may be associated with poor prognosis in patients with DM. These data may provide useful information for clinical management of patients with DM.

## 1. Introduction

Idiopathic inflammatory myopathy (IIM) is a systemic autoimmune disease that primarily affects the muscular system.^[1]^ Clinically, it is characterized by symmetrical proximal muscle weakness and pain in the limbs and can also involve other systems such as the skin, lungs, and joints, with elevated muscle enzymes being a key laboratory finding.^[1]^ IIM includes several subtypes: dermatomyositis (DM), polymyositis, anti-synthetase syndrome, inclusion body myositis (IBM), immune-mediated necrotizing myopathy, and overlap myositis.^[2]^ DM is the most common subtype of IIM, featuring characteristic rashes (e.g., Gottron’s papules, heliotrope rash, V-sign, shawl sign) with or without muscle involvement.^[2,3]^ The prevalence of DM is not well-defined, and its pathogenesis is thought to involve a combination of genetic, immune, and environmental factors.^[1]^

Interstitial lung disease (ILD) is a group of diseases primarily affecting the lung interstitium, characterized mainly by inflammation or fibrosis of the lung interstitium, with exertional dyspnea being the main symptom.^[4,5]^ ILD is a common complication of IIM, with its occurrence and severity depending on the IIM subtype, and is a major factor contributing to patients’ mortality.^[6,7]^ Previous studies indicated that ILD has the highest incidence (up to 80%) in anti-synthetase syndrome, followed by DM and polymyositis.^[6,8]^

**Figure 1.**
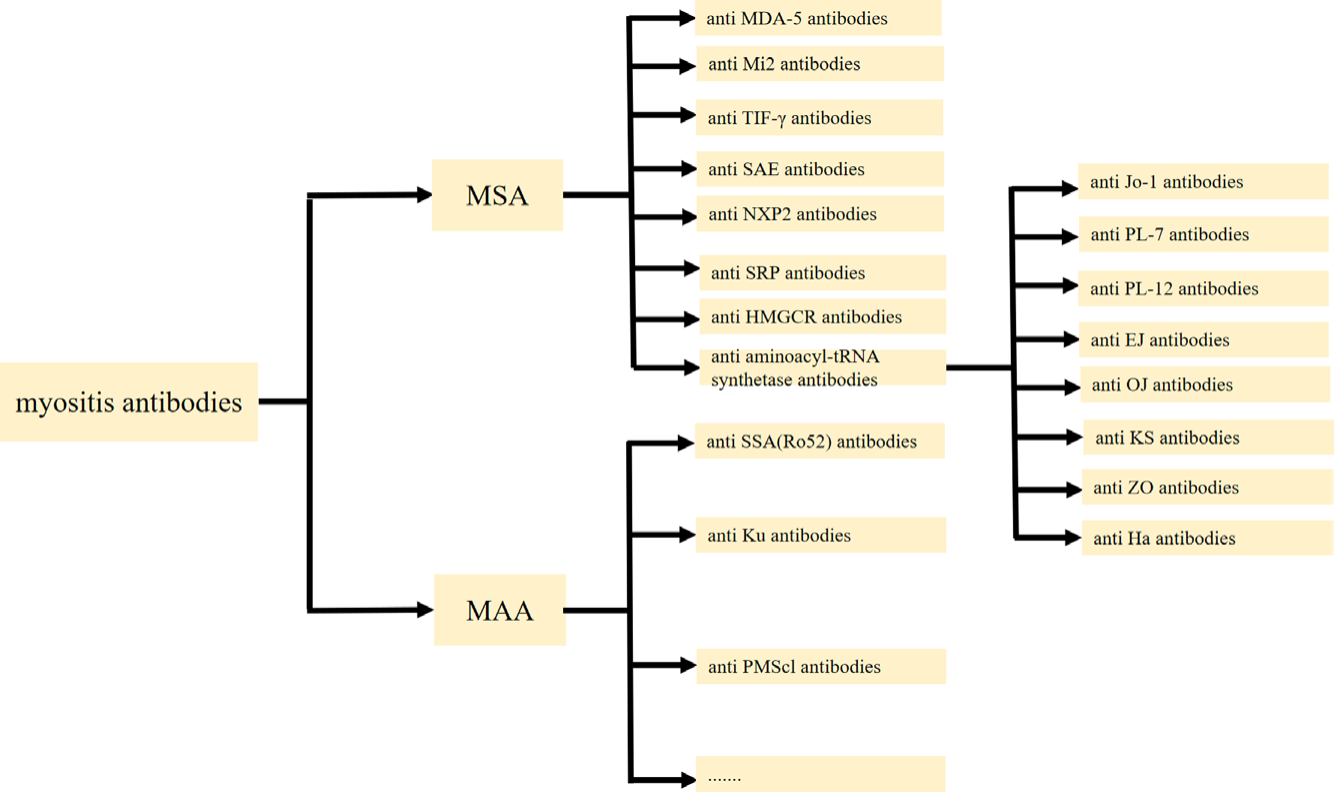
Myositis antibodies. Myositis antibodies are categorized into myositis-specific antibodies (MSA) and myositis-associated antibodies (MAA). MSA include anti-MDA-5 antibody, anti-Mi2 antibody, anti-TIF-γ antibody, anti-SAE antibody, and anti-NXP2 antibody, anti-SRP antibody, anti-HMGCR antibody, anti-aminoacyl-tRNA synthetase antibodies. MAA include anti-SSA (Ro52) antibody, anti-Ku antibody, anti-PMScl antibodies and so on.

With ongoing research into IIM, myositis-specific antibodies (MSA) and myositis-associated antibodies have become important diagnostic and prognostic tools.^[9]^ Figure 1 summarizes the autoantibodies of IIM.For DM, specific antibodies include anti-melanoma differentiation-associated gene 5 (MDA5) antibody, anti-complex nucleosome remodeling histone deacetylase (Mi2) antibody, anti-transcription intermediary factor γ (TIF-γ) antibody, anti-small ubiquitin-like modifier activating enzyme (SAE) antibody, and anti-nuclear matrix protein 2 (NXP2) antibody.^[3]^ Many studies have shown that MSA correlates closely with the clinical features and prognosis of DM.^[10–12]^ For example, anti-MDA-5 antibody is associated with ILD, especially rapidly progressive ILD and poor prognosis, while anti-TIF-γ antibody increases the risk of malignancy.^[10–12]^

The phenotype and clinical presentation of DM are complex and varied, with significant prognostic differences among phenotypes, posing challenges for the diagnosis and treatment. But most studies have focused on analyzing single antibodies. Comparative studies of DM antibody phenotypes are limited. In the present study, we hypothesized that different antibody phenotypes of DM may have different clinical characteristics. To confirm this hypothesis, we retrospectively collected DM patients from our hospital over the past 5 years and categorized these patients by MSA to analyze clinical features among different groups. The results of the present study will aid in predicting clinical outcomes and assessing prognosis for physicians.

## 2. Methods

### 2.1. Study design

**Figure 2.**
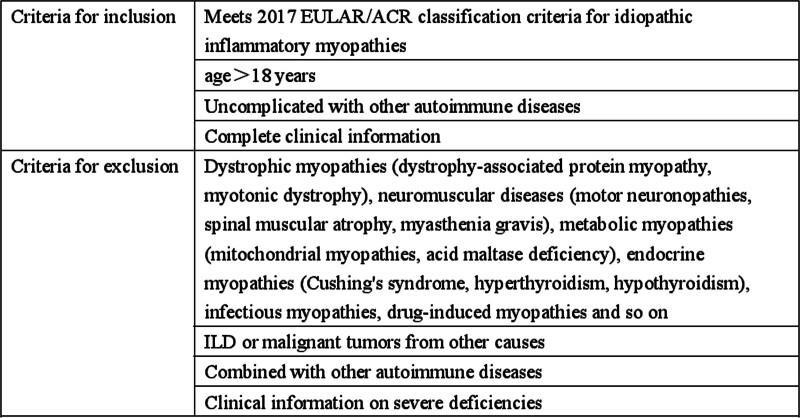
Inclusion and exclusion criteria for DM patients. The inclusion and exclusion criteria for DM patients. DM = dermatomyositis.

Our study selected DM patients admitted to Qilu Hospital of Shandong University from September 2018 to September 2023. According to the criteria shown in Figure 2, a total of 293 patients were screened, including 79 males and 214 females. Our study was conducted in accordance with the Declaration of Helsinki (2013) and was approved by the Ethics Committee of Qilu Hospital of Shandong University (Ethical approval number: KYLL-202408-037). Since this study only collected the patients’ clinical data without intervening in the diagnosis or treatment process, informed consent was waived.

### 2.2. Study factors

We used the hospital system to retrospectively collect the patients’ basic clinical data: age of onset, sex, disease duration and the presence of ILD, hypertension, diabetes, malignancies, pulmonary hypertension, pulmonary infections, and other conditions. Muscle manifestations included myasthenia, myalgia, and dysphagia. Skin manifestations included heliotrope rash, Gottron’s papules, shawl sign, V-sign, skin ulcers, mechanic’s hands, periungual erythema and Raynaud’s phenomenon. Respiratory symptoms such as dyspnea, cough and sputum and other systemic symptoms like arthralgia and fever were also recorded. Laboratory indicators included MSA, erythrocyte sedimentation rate (ESR), C-reactive protein, aspartate aminotransferase (AST), alanine aminotransferase (ALT), cardiac enzymes, complete blood count, ferritin, and immunoglobulins. Additionally, we followed up on the patients’ survival outcomes.

### 2.3. Statistical analysis

Statistical analysis was performed using SPSS 26.0 (IBM SPSS 26.0) software. Normally distributed continuous data were expressed as mean ± standard deviation ($\overset{¯}{x}\pm S$), and comparisons between groups were made using t-tests or analysis of variance (ANOVA). Skewed continuous data were expressed as median (M) (interquartile range), M (P25, P75), and comparisons between groups were made using rank-sum tests. Categorical data were expressed as counts (n) and percentages (%), and comparisons between groups were made using chi-square (χ^2^) tests or Fisher’s exact test. Differences in survival were assessed using the Kaplan–Meier survival curve and the log-rank test. Risk factors affecting mortality were analyzed using univariate and multivariate COX regression. *P* < .05 was considered a statistically significant difference.

## 3. Results

### 3.1. Clinical characterization of patients with DM

The overall mean age of onset was 52.0 ± 13.0 years in 293 patients with DM, with a median disease duration of 4 (2, 12) months, and 73% were female. As a typical feature of DM, more than half of the patients presented different rashes such as Heliotrope rash (59%), Gottron’s papule (53.9%), and V sign (57.7%). In terms of muscle involvement, myasthenia was observed in 211 patients (72%), myalgia was observed in 132 patients (45.1%), and dysphagia was observed in 58 patients (19.8%). In terms of other systemic involvement, 99 (33.8%) patients presented with arthralgia, 81 (27.6%) patients presented with fever, and 148 patients (48.5%) had combined ILD. The median values of serum creatine kinase (CK) and ESR were 103.0 (49.5, 512.5) U/L and 33 (19, 58.5) mm/h, respectively. Other specific data can be seen in Table 1.

**Table 1:** Comparison of clinical characteristics among 6 subtypes of DM patients

	All patients (n = 293)	Negative (n = 50)	MDA5 + (n = 104)	TIF1-γ+ (n = 41)	Mi2 + (n = 28)	NXP2 + (n = 19)	SAE + (n = 4)	*P* value
Age of onset (years)	52.0 ± 13.0	50.8 ± 14.5	50.7 ± 11.1	57.9 ± 12.3	54.2 ± 14.9	46.5 ± 14.6	36.0 ± 13.4	.001
Disease duration (months)	4.0 (2.0, 12.0)	6 (1, 19.5)	3 (2, 7)	6 (2.5, 21)	6 (2, 11.5)	2 (1, 24)	8 (4, 182.3)	.179
Female	214 (73.0)	38 (76)	70 (67.3)	32 (78)	22 (78.6)	11 (57.9)	3 (75)	.454
Smoking history	48 (16.4)	7 (14)	17 (16.3)	8 (19.5)	4 (14.3)	5 (26.3)	0 (0)	.810
Comorbidities								
ILD	142 (48.5)	15 (30)	84 (80.8)	7 (17.1)	12 (42.9)	5 (26.3)	1 (25)	<.001
Lung infection	80 (27.3)	10 (20)	46 (44.2)	4 (9.8)	6 (21.4)	3 (15.8)	0 (0)	<.001
Respiratory failure	14 (4.8)	1 (2)	10 (9.6)	0 (0)	1 (3.6)	0 (0)	0 (0)	.150
Tumor	29 (9.9)	7 (14)	2 (1.9)	10 (24.4)	0 (0)	1 (5.3)	0 (0)	<.001
Pulmonary arterial hypertension	17 (5.8)	3 (6)	5 (4.8)	1 (2.4)	2 (7.1)	0 (0)	0 (0)	.849
Hypertension	55 (18.8)	14 (28)	14 (13.5)	11 (26.8)	5 (17.9)	5 (26.3)	0 (0)	.181
Diabetes	46 (15.7)	7 (14)	23 (22.1)	6 (14.6)	3 (10.7)	1 (5.3)	0 (0)	.424
Skin manifestations								
Heliotrope rash	173 (59.0)	29 (58)	53 (51)	29 (70.7)	18 (64.3)	13 (68.4)	3 (75)	.254
Gottron’s papule	158 (53.9)	30 (60)	59 (56.7)	20 (48.8)	12 (42.9)	6 (31.6)	2 (50)	.261
Shawl sign	63 (21.5)	11 (22)	17 (16.3)	7 (17.1)	9 (32.1)	6 (31.6)	2 (50)	.17
V sign	169 (57.7)	35 (70)	47 (45.2)	29 (70.7)	17 (60.7)	13 (68.4)	2 (50)	.017
Skin ulcer	37 (12.6)	2 (4)	18 (17.3)	4 (9.8)	3 (10.7)	6 (31.6)	1 (25)	.031
Mechanic’s hands	20 (6.8)	3 (6)	9 (8.7)	1 (2.4)	3 (10.7)	0 (0)	0 (0)	.585
Periungual erythema	25 (8.5)	2 (4)	8 (7.7)	4 (9.8)	3 (10.7)	0 (0)	1 (25)	.341
Reynolds phenomenon	9 (3.1)	2 (4)	2 (1.9)	0 (0)	0 (0)	0 (0)	2 (50)	.007
Muscle manifestations								
Myasthenia	211 (72.0)	38 (76)	62 (59.6)	34 (82.9)	24 (85.7)	18 (94.7)	2 (50)	.002
Myalgia	132 (45.1)	25 (50)	40 (38.5)	17 (41.5)	14 (50)	16 (84.2)	0 (0)	.003
Dysphagia	58 (19.8)	10 (20)	9 (8.7)	15 (36.6)	6 (21.4)	7 (36.8)	1(25)	.001
Others								
Arthralgia	99 (33.8)	15 (30)	55 (52.9)	4 (9.8)	7 (25)	2 (10.5)	1 (25)	<.001
Fever	81 (27.6)	11 (22)	47 (45.2)	1 (2.4)	2 (7.1)	5 (26.3)	1 (25)	<.001
Pulmonary symptoms (cough/sputum/dyspnea)	122 (41.6)	13 (26)	66 (63.5)	8 (19.5)	10 (35.7)	6 (31.6)	1 (25)	<.001
Laboratory results								
ALT (U/L)	36.0 (21.0, 68.0)	34 (16.5, 50)	42 (23, 78.5)	27 (20, 35.5)	80 (36.5, 134.5)	52 (32, 90)	29 (15.5, 60.5)	<.001
AST (U/L)	45.0 (26.0, 84.5)	38.5 (20.75, 86.5)	46.5 (31.25, 66.75)	39 (24, 74.5)	119 (47, 186.75)	117 (40, 152)	35.5 (25, 73.75)	<.001
GGT (U/L)	31.5 (18.0, 65.0)	27.5 (16, 47.25)	62.5 (31, 112.75)	19 (12.5, 25.5)	18 (12.5, 37.125)	34 (24, 47)	13.5 (8, 23.5)	<.001
CK (U/L)	103.0 (49.5, 512.5)	127 (42.75, 658)	62.5 (30.25, 117.25)	158 (78, 376.5)	2123.5 (363, 3688)	1521 (413, 2606)	309.5 (56.25, 916.75)	<.001
CK-MB (ng/mL)	3.4 (1.6, 10.7)	4.3 (1.08, 17.35)	2.3 (1.3, 3.6)	5.2 (3.15, 7.85)	122.3 (3.4, 240.7)	13.8 (9.3, 25.2)	6 (2, 51.1)	<.001
CTNI (ng/L)	4.2 (1.9, 8.3)	4.15 (2.07, 6.48)	4.15 (1.87, 6.60)	3.02 (0.51, 7.39)	44.13 (4.39, 89.92)	5.59 (3.81, 9.24)	1.21 (0.09, 6.46)	<.001
LDH (U/L)	359 (277, 484)	351 (252.75, 498.75)	326.5 (277, 396.5)	336 (260, 448.5)	685.5 (394.25, 965.5)	481 (406, 610)	404.5 (263.75, 478.5)	<.001
WBC (×10^9^/L)	5.9 (4.2, 8.4)	7.40 (5.73, 8.83)	4.73 (3.46, 6.58)	5.32 (4.05, 7.92)	6.55 (4.6, 9.15)	10.17 (6.22, 12.66)	8.43 (4.83, 9.32)	<.001
NEUT (×10^9^/L)	4.3 (2.8, 6.8)	4.83 (3.88, 7.15)	3.48 (2.26, 4.94)	3.97 (2.455, 6.54)	5.09 (2.98, 7.54)	7.38 (5.09, 10.97)	5.11 (2.83, 6.73)	<.001
LYM (×10^9^/L)	0.9 (0.6, 1.3)	1.17 (0.78, 1.70)	0.76 (0.53, 1.15)	0.85 (0.69, 1.3)	0.91 (0.59, 1.25)	1.01 (0.46, 1.78)	1.59 (0.75, 2.65)	.001
EO (×10^9^/L)	0.01 (0.00, 0.06)	0.01 (0, 0.10)	0 (0, 0.04)	0.01 (0, 0.1)	0.04 (0, 0.08)	0.02 (0, 0.06)	0.13 (0.02, 0.74)	.006
BASO (×10^9^/L)	0.01 (0.01, 0.02)	0.02 (0.01, 0.03)	0.01 (0, 0.02)	0.01 (0, 0.02)	0.01 (0.01, 0.03)	0.02 (0, 0.04)	0.03 (0.02, 0.06)	.001
MONO (×10^9^/L)	0.4 (0.3, 0.6)	0.51 (0.38, 0.63)	0.36 (0.25, 0.45)	0.5 (0.34, 0.65)	0.44 (0.29, 0.55)	0.62 (0.5, 0.84)	0.68 (0.45, 0.82)	<.001
RBC (×10^12^/L)	4.2 (3.8, 4.5)	4.27 (3.85, 4.57)	4.14 (3.64, 4.43)	3.98 (3.68, 4.22)	4.33 (4.02, 4.62)	4.22 (4.01, 4.67)	4.51 (3.67, 4.64)	.011
HGB (g/L)	124 (112, 135)	126 (115.25, 137.25)	122.5 (109.25, 134)	118 (106.5, 129.5)	124.5 (110.25, 136.75)	132 (121, 139)	117.5 (103.25, 137.75)	.150
PLT (×10^9^/L)	226 (176, 283.5)	225 (184.25, 287.5)	199.5 (153.25, 273.75)	249 (182, 282.5)	275 (239.5, 337.25)	204 (170, 253)	269 (198.25, 357)	.001
CRP (mg/L)	5.1 (3.5, 7.8)	5.08 (2.01, 9.64)	5.08 (3.63, 8.27)	5.08 (5.08, 7.18)	5.08 (1.465, 5.58)	5.08 (3.12, 15.22)	14.04 (5.08, 27.5)	.413
ESR (mm/h)	33 (19, 58.5)	25.5 (18.5, 61)	40 (24.25, 66.5)	26 (14, 46.5)	29 (18.25, 61.25)	25 (12, 38)	15 (6.96, 31.25)	.005
LMR	2.1 (1.5, 3.1)	2.31 (1.59, 3.51)	2.16 (1.47, 3.03)	1.98 (1.57, 2.92)	1.96 (1.48, 3.38)	1.44 (1.07, 2.54)	1.95 (1.44, 4.55)	.254
NLR	4.5 (2.8, 7.4)	4.24 (2.63, 7.76)	4.13 (2.92, 6.55)	4.37 (2.40, 6.63)	6.08 (3.18, 8.48)	9.20 (4.30, 15.42)	3.94 (1.91, 4.80)	.019
PLR	247.9 (163.7, 353.7)	194.49 (139.99, 313.47)	268.11 (169.41, 402.74)	255.48 (185.16, 361.29)	289.35 (227.64, 515.33)	228.72 (140.87, 428.81)	224.60 (83.40, 406.23)	.031
IgG (mg/L)	13.8 (11.4, 16.3)	13.8 (10.88, 17)	14.9 (13.2, 17.58)	13.6 (11.55, 15.2)	10.9 (9.54, 13.8)	10.6 (8.44, 14.9)	15.75 (13.9, 20.98)	<.001
IgA (mg/L)	2.3 (1.8, 3.0)	2.33 (1.65, 2.93)	2.63 (2.03, 3.46)	2.22 (1.81, 2.38)	1.92 (1.54, 2.34)	1.98 (1.49, 2.23)	2.37 (2.22, 2.43)	<.001
IgM (mg/L)	1.1 (0.8, 1.4)	1.13 (0.70, 1.57)	1.10 (0.86, 1.47)	1.02 (0.72, 1.15)	0.99 (0.73, 1.18)	1.33 (0.85, 1.68)	1.19 (0.93, 1.53)	.102
C3 (g/L)	1.1 (0.9, 1.2)	1.06 (0.95, 1.18)	1.05 (0.93, 1.18)	0.99 (0.85, 1.05)	1.06 (0.95, 1.15)	1.04 (0.98, 1.16)	1.09 (1.03, 1.16)	.125
C4 (g/L)	0.3 (0.2, 0.3)	0.25 (0.19, 0.26)	0.26 (0.22, 0.32)	0.25 (0.20, 0.32)	0.25 (0.22, 0.32)	0.25 (0.2, 0.3)	0.21 (0.16, 0.25)	.106
Ferritin (μg/L)	437 (178.5, 1009.5)	317 (111.25, 613.15)	625.5 (338.25, 1196.5)	325 (100, 841.5)	234.85 (90.52, 920.7)	784 (312, 1489)	365.5 (116.33, 671.75)	<.001

*P* < .05 indicates that the difference is statistically significant.

ALT = alanine aminotransferase, AST = aspartate aminotransferase, BASO = basophil, C3 = complement 3, C4 = complement 4, CK = creatine kinase, CK-MB = creatine kinase isoenzyme, CRP = C-reactive protein, CTNI = cardiac troponin I, DM = dermatomyositis, EO = eosinophil, ESR = erythrocyte sedimentation rate, GGT = γ-glutamyl transferase, HGB = hemoglobin, IgA = immunoglobulin A, IgG = immunoglobulin G, IgM = immunoglobulin M, ILD = interstitial lung disease, LDH = lactate dehydrogenase, LMR = lymphocyte-monocyte ratio, LYM = lymphocyte, MONO = monocytes, NEUT = neutrophils, NLR = neutrophils-lymphocyte ratio, PLR = platelet-lymphocyte ratio, PLT = platelet, RBC = red blood cell, WBC = white blood cell.

### 3.2. Comparison of clinical characteristics among 6 subtypes of DM patients

After removing 35 patients who did not undergo antibody testing and 12 patients who were positive for multiple antibodies, we obtained 246 patients who were positive for a single antibody. The results were shown in Table 1. 50 (20.3%) patients were antibody negative, 104 (42.3%) patients were positive for anti-MDA5 antibody, 41 (16.7%) patients were positive for anti-TIF-γ antibody, 28 (11.4%) patients were positive for anti-Mi antibody, 19 (7.7%) patients were positive for anti-NXP2 antibody, and 4 (1.6%) patients were positive for anti-SAE antibody. Respiratory system involvement such as ILD (*P* < .001), lung infection (*P* < .001), respiratory symptoms (*P* < .001), and other systemic involvement such as: arthralgia (*P* < .001), and fever (*P* < .001) were more likely to be seen in patients with anti-MDA5 antibody, while malignancy (*P* < .001), and V sign (*P* = .017) were more likely to occur in anti-TIF1-γantibody positive patients. Anti-NXP2 antibody positive patients, on the other hand, showed more symptoms of muscle involvement, such as myasthenia (*P* = .002), myalgia (*P* = .003), and dysphagia (*P* = .001).

In terms of laboratory markers, patients with anti-Mi2 antibodies had higher myosin values [ALT (*P* < .001), AST (*P* < .001), CK (*P* < .001), creatine kinase isoenzyme (CK-MB, *P* < .001), cardiac troponin I (CTNI, *P* < .001), lactate dehydrogenase (LDH, *P* < .001)], platelet (*P* = .001), and platelet-lymphocyte ratio (PLR, *P* = .031). Anti-NXP2 antibody positive patients had higher levels of white blood cell (*P* < .001), neutrophils (*P* < .001), neutrophils-lymphocyte ratio (NLR, *P* = .019), and ferritin (*P* < .001). Anti-SAE antibody positive patients had higher levels of lymphocyte-monocyte ratio (LYM, *P* = .001), eosinophil (EO, *P* = .006), basophil (BASO, *P* = .001), monocytes (*P* < .001), red blood cell (RBC, *P* = .011), and immunoglobulin G (*P* < .001). While anti-MDA5 positive patients had higher levels of immunoglobulin A (*P* < .001), ESR (*P* = .005). Detailed data are shown in Table 1.

### 3.3. Prognostic factors of DM patients

**Figure 3.**
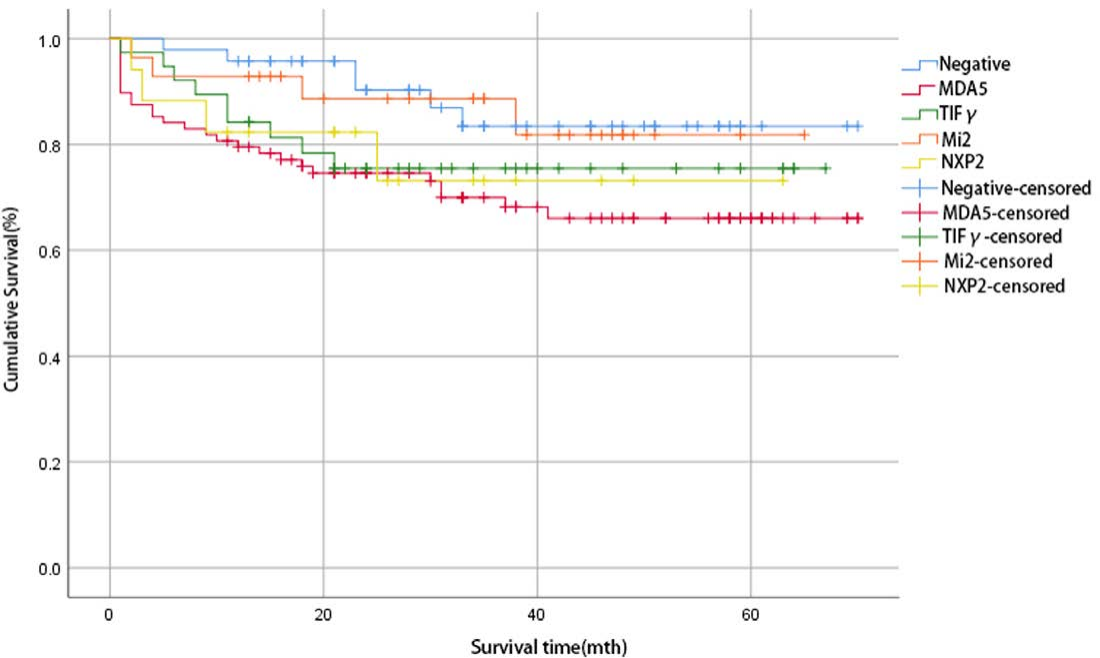
Kaplan–Meier curves of the 6 antibody subgroups. Kaplan–Meier survival curves for 6 antibody subtypes. Although the difference was not statistically significant (*P* > .05), it appeared that patients positive for anti-MDA5 antibodies had a lower survival rate, followed by anti-TIF-γ antibodies and anti-NXP2 antibodies.

During the follow-up of 293 patients, 30 patients were lost. Of the remaining 263 patients, 204 patients survived and 59 patients died, resulting in a mortality rate of 22.43%. Follow-up time ranged from (11–70) months, with a median follow-up time of 38 months. Kaplan–Meier curves for the 6 different antibody groups were shown in Figure 3. Although no statistically significant differences were observed, survival appeared to be worse in anti-MDA5 antibody-positive patients.

We also compared the baseline data between the survival and death groups. As shown in Table 2, patients in the death group had a shorter duration of illness (*P* = .009) and a higher probability of lung infection (*P* = .028), respiratory failure (*P* = .015), tumor (*P* = .018), and diabetes (*P* = .013), compared to the survival group. In terms of clinical manifestations, the death group was more likely to have fever (*P* = .015). In terms of laboratory tests, the death group had higher median levels of ALT (*P* = .009), AST (*P* < .001), γ-glutamyl transferase (GGT, *P* = .001), CTNI (*P* < .001), LDH (*P* < .001), C-reactive protein (*P* = .001), ESR (*P* = .006), NLR (*P* < .001), PLR (*P* = .002), and ferritin (*P* < .001). While the median levels of EO (*P* < .001), BASO (*P* = .012), RBC (*P* = .001), hemoglobin (*P* = .004), LYM (*P* = .001), LMR (lymphocyte-monocyte ratio, *P* < .001), complement 3 (C3, *P* = .025) were higher in the survival group.

**Table 2:** Comparison of clinical characteristics between survivors and decedents group in DM patients

	Survivors (n = 204)	Decedents (n = 59)	*P* value
Age of onset (yr)	48.6 ± 12.2	61.3 ± 11.3	.742
Disease duration (mo)	5 (2, 12)	3 (1, 6)	.009
Female	153 (75.0)	41 (69.5)	.397
Smoking history	28 (13.7)	15 (25.4)	.032
Comorbidities			
ILD	92 (45.1)	34 (57.6)	.090
Lung infection	47 (23.0)	22 (37.3)	.028
Respiratory failure	6 (2.9)	7 (11.9)	.015
Tumor	14 (6.9)	10 (16.9)	.018
Pulmonary arterial hypertension	11 (5.4)	5 (8.5)	.573
Hypertension	33 (16.2)	15 (25.4)	.105
Diabetes	25 (12.3)	15 (25.4)	.013
Skin manifestations			
Heliotrope rash	129 (63.2)	31 (52.5)	.138
Gottron’s papule	115 (56.4)	29 (49.2)	.326
Shawl sign	51 (25.0)	9 (15.3)	.116
V sign	122 (59.8)	29 (49.2)	.145
Skin ulcer	22 (10.8)	8 (13.6)	.555
Mechanic’s hands	16 (7.8)	1 (1.7)	.164
Periungual erythema	17 (8.3)	5 (8.5)	1.000
Reynolds phenomenon	6 (2.9)	1 (1.7)	.948
Muscle manifestations			
Myasthenia	144 (70.6)	47 (79.7)	.169
Myalgia	97 (47.5)	20 (33.9)	.063
Dysphagia	34 (16.7)	16 (27.1)	.072
Others			
Arthralgia	70 (34.3)	18 (30.5)	.585
Fever	47 (23.0)	23 (39.0)	.015
Pulmonary symptoms (cough/sputum/dyspnea)	83 (40.7)	24 (40.7)	.999
Laboratory results			
ALT (U/L)	32 (19.0, 63.8)	48 (25, 82)	.009
AST (U/L)	40 (24.0, 75.3)	66 (39, 116)	<.001
GGT (U/L)	25 (16.0, 54.8)	41 (29, 82)	.001
CK (U/L)	90 (47.0, 398.8)	193 (58.0, 890.0)	.077
CK-MB (ng/mL)	3.3 (1.4, 11.3)	4 (2.1, 12.0)	.160
CTNI (ng/L)	3.6 (1.6, 6.5)	7.0 (4.2, 13.4)	<.001
LDH (U/L)	327 (260.0, 465.3)	403 (348, 550)	<.001
WBC (×10^9^/L)	5.9 (4.1, 8.4)	6.1 (4.5, 8.1)	.876
NEUT (×10^9^/L)	4.2 (2.8, 6.8)	4.8 (3.6, 6.6)	.295
LYM (×10^9^/L)	1.0 (0.7, 1.4)	0.7 (0.5, 1.1)	.001
EO (×10^9^/L)	0.02 (0.00, 0.07)	0.00 (0.00, 0.02)	<.001
BASO (×10^9^/L)	0.01 (0.01, 0.02)	0.01 (0.00, 0.02)	.012
MONO (×10^9^/L)	0.4 (0.3, 0.6)	0.5 (0.3, 0.7)	.588
RBC (×10^12^/L)	4.2 (3.9, 4.5)	4.0 (3.5, 4.3)	.001
HGB (g/L)	125 (113.0, 136.8)	121 (102, 130)	.004
PLT (×10^9^/L)	234 (179.3, 278.5)	223 (181, 294)	.907
CRP (mg/L)	5.1 (2.7, 5.1)	6 (4.5, 15.2)	.001
ESR (mm/h)	29 (18.0, 52.3)	41 (24, 70)	.006
LMR	2.3 (1.7, 3.2)	1.6 (1.1, 2.1)	<.001
NLR	4.2 (2.6, 6.6)	6.1 (3.9, 9.5)	<.001
PLR	228.7 (162.3, 337.4)	313.3 (209.0, 444.1)	.002
IgG (mg/L)	13.8 (11.5, 16.4)	13.7 (10.7, 15.1)	.270
IgA (mg/L)	2.3 (1.8, 2.9)	2.3 (1.8, 2.9)	.506
IgM (mg/L)	1.1 (0.9, 1.4)	1.0 (0.7, 1.3)	.149
C3 (g/L)	1.1 (1.0, 1.2)	1.0 (0.9, 1.1)	.025
C4 (g/L)	0.3 (0.2, 0.3)	0.3 (0.2, 0.3)	.994
Ferritin (μg/L)	377.5 (130.6, 746.8)	830 (437, 1138)	<.001

*P* < .05 indicates that the difference is statistically significant.

ALT = alanine aminotransferase, AST = aspartate aminotransferase, BASO = basophil, CK = creatine kinase, CK-MB = creatine kinase isoenzyme, C3 = complement 3, C4 = complement 4, CRP = C-reactive protein, CTNI = cardiac troponin I, DM = dermatomyositis, EO = eosinophil, ESR = erythrocyte sedimentation rate, GGT = γ-glutamyl transferase, HGB = hemoglobin, IgA = immunoglobulin A, IgG = immunoglobulin G, IgM = immunoglobulin, ILD = interstitial lung disease, LDH = lactate dehydrogenase, LMR = lymphocyte-monocyte ratio, LYM = lymphocyte, MONO = monocytes, NEUT = neutrophils, NLR = neutrophils-lymphocyte ratio, PLR = platelet-lymphocyte ratio, PLT = platelet, RBC = red blood cell, WBC = white blood cell.

To further analyze the factors affecting the prognosis of DM patients, we first used the one-way COX proportional risk model to screen out variables that might have an impact on prognosis. Then, we incorporated these variables to construct a multifactorial COX proportional risk model. The results revealed that: age at onset (hazard ratio = 1.096, 95% CI: 1.064–1.129, *P* < .001), fever (hazard ratio = 2.449, 95% CI: 1.183–5.066, *P* = .016), GGT level (hazard ratio = 1.005, 95% CI: 1.002–1.008, *P* < .001), EO level (hazard ratio = 0.000, 95% CI: 0.000–0.324, *P* = .024), and C3 level (hazard ratio = 0.115, 95% CI: 0.023–0.575, *P* = .008) had a statistically significant effect on survival time (Table 3).

**Table 3:** Multivariate Cox regression analysis of prognostic factors in patients with DM

Variables	β	SE	*Z*	*P*	Hazard ratio (95% CI)
Age of onset (yr)	0.092	0.015	6.025	<.001	1.096 (1.064–1.129)
Smoking history	0.542	0.344	1.575	.115	1.719 (0.876–3.371)
ILD	0.525	0.361	1.454	.146	1.691 (0.833–3.433)
Lung infection	0.064	0.344	0.187	.852	1.066 (0.543–2.095)
Respiratory failure	0.942	0.499	1.888	.059	2.564 (0.965–6.814)
Hypertension	0.124	0.376	0.330	.741	1.132 (0.542–2.365)
Diabetes	−0.363	0.361	−1.007	.314	0.695 (0.343–1.411)
Tumor	0.440	0.423	1.039	.299	1.553 (0.677–3.559)
Myalgia	0.128	0.331	0.386	.699	1.137 (0.594–2.176)
Dysphagia	0.443	0.368	1.206	.228	1.558 (0.758–3.202)
Fever	0.895	0.371	2.414	.016	2.449 (1.183–5.066)
ALT (U/L)	−0.002	0.003	−0.625	.532	0.998 (0.991–1.005)
AST (U/L)	0.002	0.002	0.994	.320	1.002 (0.998–1.007)
GGT (U/L)	0.005	0.001	3.706	<.001	1.005 (1.002–1.008)
LDH (U/L)	0.001	0.001	0.873	.383	1.001 (0.999–1.002)
EO (×10^9^/L)	−8.557	3.791	−2.257	.024	0.000 (0.000–0.324)
BASO (×10^9^/L)	−5.194	12.565	−0.413	.679	0.006 (0.000–275378602.827)
RBC (×10^12^/L)	0.395	0.561	0.704	.481	1.485 (0.494–4.463)
HGB (g/L)	−0.012	0.019	−0.617	.537	0.988 (0.952–1.026)
ESR (mm/h)	−0.007	0.005	−1.311	.190	0.993 (0.982–1.004)
NLR	−0.011	0.031	−0.354	.724	0.989 (0.931–1.051)
PLR	0.001	0.001	1.007	.314	1.001 (0.999–1.003)
C3 (g/L)	−2.165	0.822	−2.633	.008	0.115 (0.023–0.575)

*P* < .05 indicates that the difference is statistically significant.

ALT = alanine aminotransferase, AST = aspartate aminotransferase, BASO = basophil, C3 = complement 3, DM = dermatomyositis, EO = eosinophil, ESR = erythrocyte sedimentation rate, GGT = γ-glutamyl transferase, HGB = hemoglobin, ILD = interstitial lung disease, LDH = lactate dehydrogenase, NLR = neutrophils-lymphocyte ratio, PLR = platelet-lymphocyte ratio, PLT = platelet, RBC = red blood cell.

## 4. Discussion

As one of the most common subtypes of IIM, DM has received much attention, especially with its associated MSA.^[13]^ In current diagnostic criteria, neither the 1975 Bohan and Peter’s classification criteria^[14,15]^ nor the 2017 EULAR (European League Against Rheumatism)/ACR (American College of Rheumatology) classification criteria on IIM^[16]^ include DM-associated MSA. Therefore, we analyzed the clinical characteristics between the 6 DM-specific antibody subgroups and between the survival and mortality groups, as well as the risk factors affecting the prognosis of DM, combining previous studies for in-depth discussion.

In this study, the mean age of onset of DM in adults was around 52 ± 13 years, with a female:male ratio of approximately 2.7:1, which was slightly higher than previous studies.^[3,17]^ Skin changes were key to the diagnosis of DM, and common skin changes included heliotrope rash (purplish-red edematous erythema distributed symmetrically in the periorbital area), gottron’s papule (rash located on the extensor surfaces of the joints), V-sign (erythema in the V region of the cervico-thoracic area), and shawl sign (erythema distributed on the upper back and deltoid muscles), mechanic’s hands (hyperkeratosis of the fingers), and skin ulcer.^[3]^ In our study, we found that more than half of the patients with DM presented with heliotrope rash (59%), gottron’s papule (53.9%), V-sign (57.7%), and other skin changes such as shawl sign (21.5%), skin ulcer (12.6%), mechanic’s hands (6.8%), periungual erythema (8.5%), and reynolds phenomenon (3.1%) can also be found in DM patients, but are not as representative as the former.

According to previous studies, there are 5 types of MSA associated with DM, namely anti-MDA5 antibody, anti-TIF-γ antibody, anti-Mi2 antibody, anti-NXP2 antibody, and anti-SAE antibody, and each antibody has its own unique clinical characteristics.^[18]^ Among our collected patients, 20.3% were antibody-negative, 42.3% were positive for anti-MDA5 antibodies, 16.7% were positive for anti-TIF-γ antibodies, 11.4% were positive for anti-Mi2 antibodies, 7.7% were positive for anti-NXP2 antibodies, and 1.6% were positive for anti-SAE antibodies. Anti-MDA5 antibodies are relatively common in DM patients, accounting for approximately 1% to 30% of DM, which is slightly lower than our data.^[19]^ More than half of anti-MDA5 antibody-positive patients develop ILD, including rapidly progressive ILD, a subtype that can lead rapidly progressive respiratory deterioration with a very high mortality rate.^[20]^ Compared with other types of antibodies, anti-MDA5 antibody-positive patients developed skin or oral ulcers, mechanic’s hands, arthralgia and fever more frequently. Therefore, the systemic inflammatory response in anti-MDA5 antibody-positive patients was more severe and had a poorer prognosis.^[10,19,20]^ Consistent with the results of previous studies, our study demonstrated that patients positive for anti-MDA5 antibodies were more likely to develop ILD, lung infections, respiratory symptoms, arthralgia, and fever, and the inflammatory index ESR was relatively high compared to other types of antibodies. Meanwhile, among the 59 patients who died, there were 27 patients with anti-MDA5 antibody accounting for almost half of them. This suggested that patients with anti-MDA5 antibodies had an extremely poor prognosis. But unlike previous studies, our study found that anti-NXP2 positive patients were more likely to develop skin ulcers. Many studies have demonstrated that anti-TIF-γ antibodies are associated with severe rashes and malignant tumors, and their susceptibility to malignancy may be due to the fact that it is an inhibitor of tumor necrosis factor.^[13,21]^ In our study, we found that 24.4% of anti-TIF-γ antibody-positive patients were comorbid malignant tumors, which is much higher than other types of antibodies. As for rash, we only found that anti-TIF-γ antibody positive patients were more likely to have V sign. Tanboon et al^[22]^ and Pinal-Fernandez et al^[23]^ found that anti-Mi-2 antibodies caused greater damage to the muscle, associated with high levels of CK. Our study also found elevated muscle enzyme values [ALT (*P* < .001), AST (*P* < .001), CK (*P* < .001), CK-MB (*P* < .001), CTNI (*P* < .001), and LDH (*P* < .001)] in patients with anti-Mi antibody positivity which were significantly higher than the other subtypes, and the occurrence of symptoms of muscle involvement such as myalgias and myalgia was also high, second only to anti-NXP2 antibody. Anti-NXP2 antibodies are the most common antibodies in young DM patients. Several studies have shown that anti-NXP2 antibodies are associated with peripheral edema, muscle weakness, myalgia, dysphagia, calcinosis, and rash, and have a favorable prognosis.^[24–26]^ Our study only found that patients positive for anti-NXP2 antibodies showed more symptoms of muscle involvement such as muscle weakness (*P* = .002), muscle pain (*P* = .003), dysphagia (*P* = .001), and a lower probability of comorbid rashes than other antibodies. Since anti-SAE antibodies are less frequently seen in DM patients, limited studies have been conducted both nationally and internationally.^[27]^ Some studies have found that anti-SAE antibodies are associated with a mild typical rash and muscle involvement, but a favorable prognosis is the consensus.^[28–30]^ Our study found that anti-SAE antibody-positive patients had higher levels of LYM (*P* = .001), EO (*P* = .006), BASO (*P* = .001), monocytes (*P* < .001), RBC (*P* = .011), and immunoglobulin G (*P* < .001), and we found no peculiarities in terms of clinical symptoms, which might be related to our sample size was too small. The sample size of patients with anti-SAE antibodies in this study was small but also represented a certain clinical profile. Broader and more generalized clinical features were required in further study.

We also analyzed the prognosis of patients with DM. 59 of the 263 patients followed up were died, with a mortality rate of 22.43%. In addition to combined with lung disease, tumors, elevated muscle enzymes and inflammatory factors, which have been demonstrated many times.^[31]^ In a comparison between the survival and death groups, we found that patients in the death group were more likely to have fever, elevated NLR, PLR, and ferritin levels. NLR is the ratio of neutrophils to lymphocytes in peripheral blood, and previous studies had shown that it is associated with an increased risk of death in patients with DM, and elevated serum ferritin levels had also been associated with disease severity and poor prognosis.^[19,30]^ Survival curves had shown that patients who were positive for anti-MDA5 antibodies had a reduced survival time compared to other types of antibodies. It has been shown that high age, male gender, comorbid lung diseases and tumors, serum ferritin, and NLR levels are factors that influence the prognosis of DM patients.^[30–32]^ After implementing a multifactorial COX regression analysis, we found that increased age at onset (hazard ratio = 1.096, 95% CI: 1.064–1.129, *P* < .001), combined fever (hazard ratio = 2.449, 95% CI: 1.183–5.066, *P* = .016), increased GGT levels (hazard ratio = 1.005, 95% CI: 1.002–1.008, *P* < .001), and decreased C3 (hazard ratio = 0.115, 95% CI: 0.023–0.575, *P* = .008), and EO levels (hazard ratio = 0.000, 95% CI: 0.000–0.324, *P* = .024) were risk factors for death in DM patients.

There are some limitations of this study. Firstly, due to the regional and resource constraints, we were only able to collect research resources from Chinese in our center, which may introduce selection bias. At the same time, however, a number of measures had been taken to minimize bias, such as the inclusion of more comprehensive indicators and the use of objectively recorded data. Secondly, partial data were lost which did not affect the determination of the overall clinical features of the disease. All important indicators that were representative of clinical features were tested. These indicators were necessary to guide clinical treatment and judgment of the condition. Thirdly, genetic, environmental and lifestyle factors can influence patients with DM in varying degrees, but there was no comprehensive information on the above for patients attending our clinic for routine treatment. It was of clear clinical significance that by focusing on the antibody phenotype as an important indicator of prognosis and treatment. At the same time, the condition can be effectively determined in real time and treatment can be guided. Finally, the relationship between GGT, C3, and EO levels and DM patients was less frequently included in studies, so more subsequent studies are needed to confirm it.

In conclusion, DM is a complex disease that can be categorized into different subtypes based on MSA. Understanding the clinical features possessed by each subtype can be helpful in predicting the patient’s clinical presentation, response to treatment, and assessing prognosis.

## Author contributions

**Conceptualization:** Xiaowen Xie.

**Data curation:** Xinyue Dai.

**Formal analysis:** Xiaowen Xie.

**Investigation:** Xinyue Dai.

**Methodology:** Xiaowen Xie, Yi Xing.

**Project administration:** Huaxiang Liu, Yi Xing.

**Writing – original draft:** Xiaowen Xie.

**Writing – review & editing:** Huaxiang Liu, Yi Xing.
